# Top‐predator carrion is scary: Fight‐and‐flight responses of wild boars to wolf carcasses

**DOI:** 10.1002/ece3.9911

**Published:** 2023-04-05

**Authors:** Daniel Redondo‐Gómez, Luca Rossi, Mattia Cardello, Soraya De Pasquale, Carlos Martínez‐Carrasco, José A. Sánchez‐Zapata, Marcos Moleón

**Affiliations:** ^1^ Department of Zoology University of Granada Granada Spain; ^2^ Department of Veterinary Sciences University of Torino Torino Italy; ^3^ Department of Animal Health University of Murcia Murcia Spain; ^4^ Department of Applied Biology University Miguel Hernández Elche Spain

**Keywords:** antipredator responses, *Canis lupus*, landscape of fear, predation risk, predator avoidance, prey behavior

## Abstract

Predation risk largely constrains prey behavior. However, whether predators may be scary also after death remains unexplored. Here, we describe the “fight‐and‐flight” responses of a prey, the wild boar (*Sus scrofa*), to carcasses of (a) its main predator, the gray wolf (*Canis lupus*) and (b) a carnivore that very rarely kills wild boars, the red fox (*Vulpes vulpes*), in the western Alps (Italy). We recorded the behavior of wild boars at 10 wolf and 9 fox carcass sites. We found eight “fight‐and‐flight” responses toward wolf carcasses, and none toward fox carcasses. Our results suggest that carnivore carcasses may indeed be scary; fear responses toward them are dependent on the species to which the carcass belongs; and animals approaching the carcasses are feared mainly when the latter are relatively fresh. This emphasizes the multiple and complex roles that carrion plays in the landscape of fear and opens exciting ecological, epidemiological, and evolutionary research avenues.

## Graphical abstract

Predation risk largely constrains prey behavior. However, whether predators may be scary also after death remains unexplored. Here, we describe the ‘fight‐and‐flight’ responses of a prey, the wild boar, to carcasses of its main predator, the gray wolf.

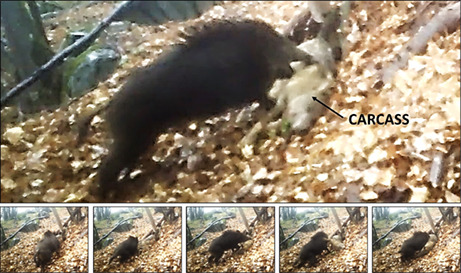

## 1 INTRODUCTION

Predation risk greatly shapes prey's use of space and time (Valeix et al., 2009). Predator–prey coevolution has led to the refinement of defensive behaviors in prey to avoid (pre‐encounter responses) and escape (postencounter responses) predators (Mitchell, 2009). The risk of being attacked is generally higher around resources that attract predators, such as surface water (Owen‐Smith, 2020). The ‘landscape of fear’ resulting from the assessment of these resources and their associated risks may help animals to avoid dangerous situations (Gaynor et al., 2019; Laundré et al., 2010). However, prey failing to recognize these resources—or prey that need to use the same resources as predators—may suffer a higher risk of encountering, and therefore facing, predators.

Carrion resources greatly shape the prey's landscape of fear through two main ways (Moleón & Sánchez‐Zapata, 2021). On one hand, many scavengers are also predators (Moleón et al., 2014), which may lead to increased predation risk around carcass sites to not only herbivores (Frank et al., 2020), but also subordinate predators (Atwood & Gese, 2008). As a response, some prey seem to avoid carcass sites (Cortés‐Avizanda et al., 2009). On the other hand, a fresh carcass, especially that of a predator, can also be scary in itself, as the animals approaching it may not be sure if the animal is dead or just sick, injured, or asleep, in which case it could turn on them. However, the scientific community has just started to uncover how animals respond to carnivore carcasses (e.g., Gonzálvez, Martínez‐Carrasco, & Moleón, 2021; Gonzálvez, Martínez‐Carrasco, Sánchez‐Zapata, et al., 2021), probably due in part to the difficulties in obtaining this type of carcass. In particular, the extent to which dead carnivores may frighten remains virtually unexplored to date. Here, thanks to an intensive monitoring program of apex predator carrion, we describe the first recorded observations of “fight‐and‐flight” responses (Cannon, 1915) of a prey, the wild boar (*Sus scrofa*), to carcasses of its main predator, the gray wolf (*Canis lupus*). In our context, “fight responses” occur when the animal attacks the carcass, and “flight responses” occur when the animal quickly changes the direction of its movement and immediately runs away far from the carcass site.

## 2 METHODS

In April 2022, we started the systematic monitoring of wolf carcasses by camera trapping in the high Susa Valley (western Alps, Italy). This area (600 km^2^; altitude: 600–3500 m a.s.l.) is characterized by a typical xeric inner‐alpine climate. After the eradication of the local wolf population in the 1920s (Zimen & Boitani, 1975), wolves spontaneously recolonized the area in the 1990s (Bertotto et al., 2020; Marucco & McIntire, 2010), having re‐established a viable reproductive wolf population (Marucco & Avanzinelli, 2018). In the last years, several tens of wolves are annually roadkilled in this area and the surrounding valleys. Recovered carcasses are gathered by the Department of Veterinary Sciences of the University of Torino for necropsy and preservation at −20°C. These carcasses represent a rare opportunity to regularly monitor the ecological role of this apex carnivore's carrion. In the first round of monitoring, we deployed 10 wolf carcasses (one of them being a suspected *C. lupus* × *C. familiaris* hybrid) across the valley. As a control, we used carcasses (*n* = 9) of a mesocarnivore, the red fox (*Vulpes vulpes*), which may scavenge though very rarely predates upon adult wild boars (Balestrieri et al., 2011). During ca. 40 days (period after which the carcasses were mostly composed of skin, bones, and other hard tissues), we recorded the behavior of animals approaching these carcasses using 10‐ to 60‐s videos (taken after detection of movement; interval between consecutive videos: 1 min; for more details, see Gonzálvez, Martínez‐Carrasco, & Moleón, 2021; Gonzálvez, Martínez‐Carrasco, Sánchez‐Zapata, et al., 2021). Images recorded by the cameras were grouped into “events”, that is, groups of consecutive videos of individuals of the same species taken more than 30 min apart (Gonzálvez, Martínez‐Carrasco, Sánchez‐Zapata, et al., 2021). The altitude of carcass sites was 808–1942 m, and the mean distance between neighboring carcasses was 2.1 km (range: 1–5.25 km). All carcasses were placed within conifer (mostly, *Picea abies* and *Pinus sylvestris*) and broad‐leaved (mostly, *Quercus robur* and *Fagus sylvatica*) forests, which are the main wolf habitats in the study area. In these forests, wild boars and other ungulates (red deer *Cervus elaphus* and roe deer *Capreolus capreolus*) are abundant. Wild boars are commonly preyed on by wolves in the western Alps and other nearby mountain systems (Meriggi et al., 2011; Mori et al., 2017; Poulle et al., 1997).

## 3 RESULTS

In total, wild boars appeared in 26 and 14 events at 8 wolf and 6 fox carcasses, respectively. We recorded six fear‐related events at wolf carcass sites (23% of total events at wolf carcass sites) and none at fox carcass sites. These fear‐related events included two fight and six flight responses performed by at least five different adult wild boars towards three different wolf carcasses (i.e., 30% of monitored wolf carcasses). Most of these fear responses were recorded during the first stages of the decomposition process (seven responses recorded 2–3 days after carcass deployment and one registered 14 days after carcass deployment) in three different videos. Piglets were recorded at both wolf and fox carcasses.

**FIGURE 1:**
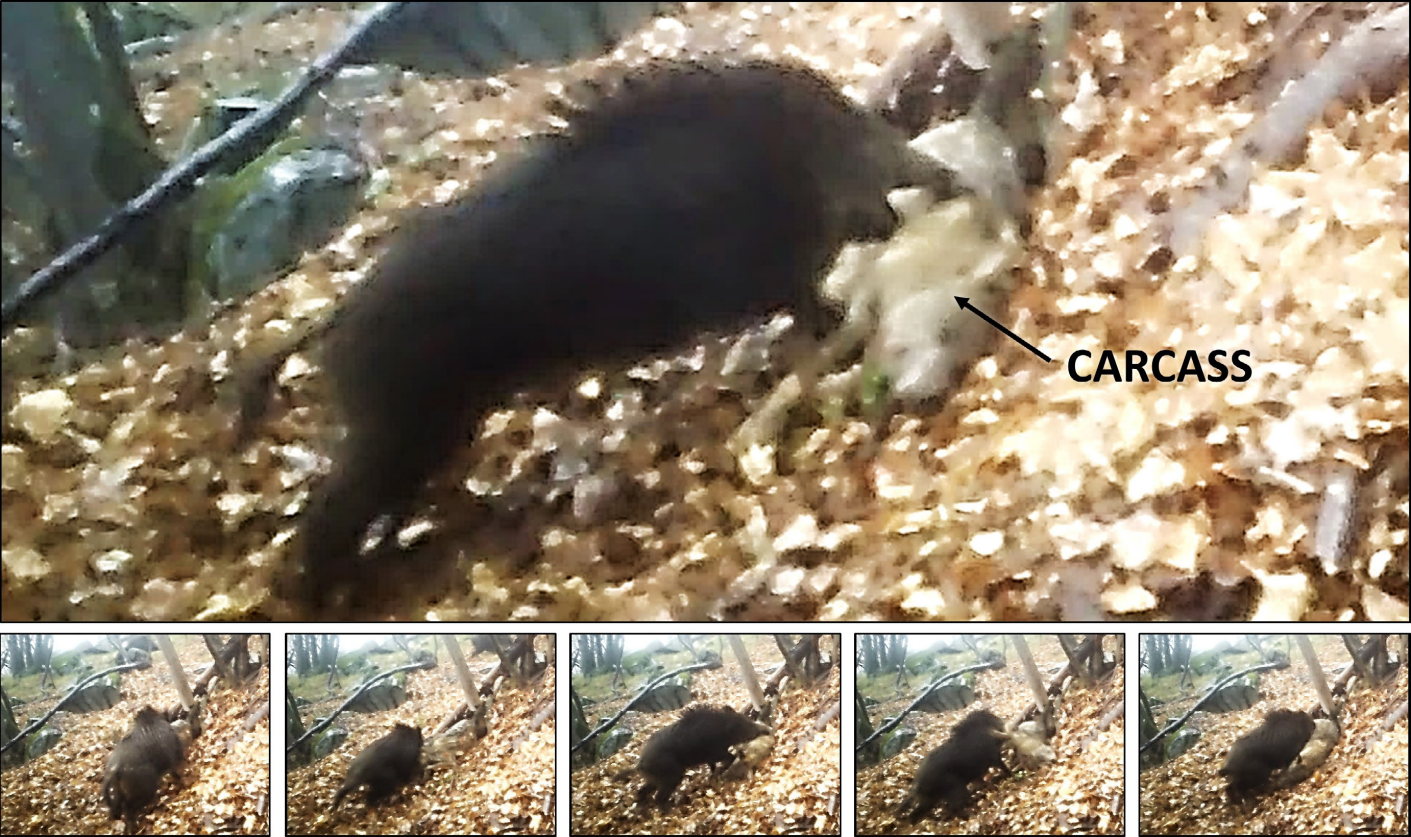
Frames of a video recorded by a camera trap at a gray wolf carcass in Susa Valley, western Alps (Italy). Images show fight‐and‐flight responses of a wild boar, 3 days after carcass deployment (April 23, 2022, at 6:13 p.m.). Frames are chronologically arranged from left to right and the upper image shows an in‐between moment in which the wild boar bites the wolf carcass.

The two fight responses to a wolf carcass were recorded in one single video on April 23 at 6:13 p.m., under slightly foggy conditions (see Figure 1; Video S1). The video starts by showing an adult‐sized wild boar (probably a female) approaching the carcass. Then, the wild boar detects it and stops. After a few seconds of hesitation from a distance to the carcass, the wild boar starts ramming it very aggressively, while strongly grunting. It rams twice the wolf's abdomen, lifting it off the ground. Then, the wild boar bites the wolf's hind leg and shakes the carcass vigorously. After this first attack, the wild boar flees and another adult‐sized member (also probably a female) of its same group starts attacking the carcass, including biting, charging, and shaking it. During this second attack, some piglets approach the carcass, along with other adult‐sized boars, which keep grunting. Finally, the group progressively leaves the scene, performing several flight responses (see Video S1). The wild boar group consisted of at least four adult‐sized individuals (probably all females, all of them with the dorsal hairs standing on end during the event) and five piglets. The video was taken 3 days after the carcass deployment, and no wild boar was recorded by the camera after this event.

## 4 DISCUSSION

Our results suggest that (a) carnivore carcasses may indeed be scary, (b) fear responses toward them are dependent on the species to which the carcass belongs, with a distinction between potential predators and nonpredators of the visitor species, and (c) animals approaching the carcasses are feared mainly when the latter are relatively fresh, and thus uncertainty about the state of the carcass (i.e., whether dead or alive) is greater. Future research is needed to explore the generality of our findings, including the responses of other scavenger and nonscavenger species visiting the carnivore carcass sites. In the case of the wild boar and other social species, whether group size and the presence of newborn individuals (see Video S1) may affect the frequency and aggressiveness of their fear responses, requires further investigation. In general, a closer examination of the use of space and time by prey species could reveal the extent to which their spatiotemporal behavior is modulated by direct encounters with predator carcasses. In our case, after fighting the wolf carcass, no wild boars were observed around the carcass for at least 5 weeks.

Overall, our findings highlight that predators can shape their prey's behavior not only while they are alive but also after their death. This both emphasizes and expands the complexity and dynamism of the landscape of fear, as well as the multiple roles that carrion plays upon it (Moleón & Sánchez‐Zapata, 2021, 2022). Acknowledging that carcasses may either directly (present study) or indirectly (e.g., by gathering scavenging predators) influence the predation risk that prey perceive opens exciting ecological, epidemiological, and evolutionary research avenues.

## AUTHOR CONTRIBUTIONS

**Daniel Redondo‐Gómez:** Conceptualization (equal); data curation (lead); formal analysis (equal); investigation (equal); methodology (equal); visualization (lead); writing – original draft (lead). **Luca Rossi:** Conceptualization (equal); investigation (equal); methodology (equal); resources (equal); supervision (equal); visualization (equal); writing – original draft (equal). **Mattia Cardello:** Investigation (equal); methodology (equal); writing – original draft (equal). **Soraya De Pasquale:** Investigation (equal); methodology (equal); writing – original draft (equal). **Carlos Martínez‐Carrasco:** Conceptualization (equal); investigation (equal); methodology (equal); resources (equal); supervision (equal); visualization (equal); writing – original draft (equal). **Jose A. Sanchez‐Zapata:** Conceptualization (equal); investigation (equal); methodology (equal); resources (equal); supervision (equal); visualization (equal); writing – original draft (equal). **Marcos Moleón:** Conceptualization (equal); funding acquisition (lead); investigation (equal); methodology (equal); project administration (lead); resources (lead); supervision (lead); visualization (equal); writing – original draft (equal).

## ACKNOWLEDGMENTS

This study was funded by MCIN/AEI/10.13039/501100011033 and “ERDF A way of making Europe” through project PID2021‐128952NB‐I00. DRG was funded by a predoctoral grant from the Junta de Andalucía (PREDOC_00262). MM was partly supported by a research contract Ramón y Cajal from the MINECO (RYC‐2015‐19231).

## FUNDING INFORMATION

This study was funded by MCIN/AEI/10.13039/501100011033 and “ERDF A way of making Europe” through project PID2021‐128952NB‐I00. DRG was funded by a predoctoral grant from the Junta de Andalucía (PREDOC_00262). MM was partly supported by a research contract Ramón y Cajal from the MINECO (RYC‐2015‐19231).

## Supporting information


Video S1

## Data Availability

Data will be provided at Dryad for peer review and will be permanently archived upon publication.
